# Editorial: Ion homeostasis in plant stress and development, volume II

**DOI:** 10.3389/fpls.2023.1264817

**Published:** 2023-08-11

**Authors:** José M. Mulet, Francisco Campos, Lynne Yenush

**Affiliations:** ^1^Instituto de Biología Molecular y Celular de Plantas (IBMCP), Universitat Politècnica de València-Consejo Superior de Investigaciones Científicas, Valencia, Spain; ^2^Departamento de Biología Molecular de Plantas, Instituto de Biotecnología, Universidad Nacional Autónoma de México, Cuernavaca, Morelos, Mexico

**Keywords:** ionome, rice, poplar, salt stress, carotenoids

## Introduction

Anthropogenic global warming is increasing the aridity and salinity of most soils, and having a great impact in natural environments (Taïbi et al., 2015) and in cultivated land (Melino and Tester, 2023). Maintaining ion homeostasis is a fundamental requirement for all living organisms, particularly important for plants, given their sessile nature that makes them vulnerable to fluctuations in soil composition and changes in salinity. Correct ion homeostasis is essential to enable all biochemical processes while preventing toxic accumulation of minerals, mainly sodium and heavy metals (Flowers and Colmer, 2015). Living organisms have evolved efficient systems to acquire, store, and regulate mineral concentrations within specific physiological ranges to ensure normal development. Maintaining ion homeostasis under stress conditions consumes substantial energy, which can hamper plant development and compromise adaptation to new environments and competition for ecological niches. Therefore, the joint regulation of ion homeostasis and metabolic pathways related to growth and development is a major question to understand how energy is diverted from some pathways to the others, how this affects plant growth and yield, and to develop novel biotechnological applications (Bromham et al., 2020).

In our previous Research Topic, we focused on the relationship between ion transport and drought stress, hormone regulation and calcium and phosphate homeostasis (Mulet et al., 2020). In the studies collected in this Research Topic, we have broadened the scope and gained novel insights into this pivotal topic for plant molecular biology and biotechnology, in both the model plant *Arabidopsis thaliana*, as well as crop plants:

## Potassium and cesium transport regulation in Arabidopsis

Arabidopsis is not only a good model system for gaining basic knowledge of plant molecular biology, but also for applied science and the development of applications that may be transferred to crops (Benito et al., 2022). Potassium is absorbed by roots and then loaded into the xylem to be transported to the aerial part of the plant. Xylem loading is an essential point in potassium homeostasis that depends on the SKOR protein. High-affinity potassium uptake by the root relies on the member of the HAK/KUP/KT transporter family HAK5 (Mostofa et al., 2022). The paper by Kanno et al. performs very precise measures of potassium and the potassium analogue cesium transportation using radioactive isotopes in wild-type Arabidopsis and in *skor* mutants. The main conclusion is that high-affinity potassium transport in the root is regulated not only by external potassium concentration; internal potassium levels also regulate HAK5 expression. Authors show that HAK5 is also responsible for cesium uptake, but the pattern of accumulation and distribution is different, indicating that once inside the plant, the affinity of different ion transporters for cesium and potassium may be different.

## Ion homeostasis in rice

Rice is the main staple crop for 25% of the world’s population (Badoni et al., 2023). Therefore, any advance in the mechanism of ion homeostasis regulation may be of great interest and are likely to lead to novel developments for improving world nutrition. In this Research Topic, we present two interesting advances in our knowledge of rice molecular biology. Wang et al. identify and characterize a novel participant in potassium uptake in roots. Using CRISPR/Cas9-mediated mutagenesis, they have disrupted the OsHAK8 gene and found that the plant becomes very sensitive to low K+ conditions. Authors show that the gene is expressed in many root cell types and targeted to the plasma membrane, so authors propose that OsHAK8 may be important not only for high-affinity potassium uptake in roots but also for root to shoot translocation. On the other hand, Zhang et al. characterize another gene from the HAK/KUP/KT family: OsHAK12, with very interesting results. Unlike other members of the family, it is permeable to sodium, upregulated by salt stress, and mainly expressed in root vascular tissue. Taken together, the authors propose that the main function of OsHAK12 is to exclude sodium from the shoot under salt stress conditions.

## Copper nutrition in woody plants

Copper is an essential micronutrient for plants, as it participates in the metallic core of several enzymes, but it can also lead to the formation of hydroxyl radicals (Mir et al., 2021). Therefore, copper homeostasis should be tightly controlled, and the window between copper nutrition and copper toxicity is very narrow. Hunter et al. provide an original approach to this problem by studying the interplay among copper homeostasis and other components of the ionome in poplar and how it affects different organs of the plant. According to their results, copper deficiency alters ion composition in leaves, while nutrient partitioning in stems is influenced mainly by the age of the plant. Their work unveils an unexpected interplay among copper nutrition, ion distribution, and plant aging.

## Intracellular phosphate homeostasis and carotenoids

Breeding for high carotenoid-containing plants is a strategy to provide healthier food. Carotenoid biosynthesis is dependent on phosphate availability as glyceraldehyde-3-phosphate is a precursor for its biosynthesis (Stra et al., 2023). The review of Hao et al., included in the Research Topic summarizes the knowledge on all the phosphate transporters localized in plastids, and focuses on their relation to carotenoid accumulation, summarizing the published information.

## Outlook

This Research Topic has provided new and valuable information regarding the differential function and regulation of three HAK proteins (HAK5, HAK8, and HAK12), the influence of copper nutrition on the ionome of woody plants, and a review on phosphate transporters as a target to enhance carotenoid accumulation. It becomes crystal-clear that ion homeostasis cannot be studied as an isolated process, but must be considered in combination with essential processes of plant physiology regarding nutrition, growth, development, and stress response pathways. These studies provide novel and valuable information on the crosstalk of ion homeostasis with other essential processes, not only in model plants, but also in crops and woody plants. We hope that future investigation will convert this information into improved crops with enhanced tolerance to abiotic stress or enhanced nutritional content.

## Author contributions

JM: Writing – original draft. FC: Writing – review & editing. LY: Writing – review & editing.
